# Jejunogastric intussusception presenting as tumor bleed

**DOI:** 10.4103/0974-2700.70775

**Published:** 2010

**Authors:** Shiraz Ahmad Rather, Tanveer Iqbal Dar, Rauf A Wani, Asima Khan

**Affiliations:** Department of General Surgery, Sher-i-Kashmir Institute of Medical Sciences, Srinagar, Jammu and Kashmir – 190 011, India

**Keywords:** Gastrectomy, jejunogastric, intussusception

## Abstract

Jejunogastric intussusception (JGI) is a rare but serious complication of previous gastrectomy or gastrojejunostomy, and a delayed diagnosis can lead to catastrophe. It can present as hematemesis, and an endoscopist aware of the condition can diagnose it early. We present a case of JGI presenting as hematemesis and diagnosed as tumor bleed on endoscopy. Diagnosis of JGI was confirmed on laparotomy, gangrenous efferent limb was resected and a fresh gastrojejunostomy performed.

## INTRODUCTION

Jejunogastric intussusception (JGI) is a rare complication of partial gastrectomy or gastrojejunostomy with no medical treatment,[1] and it can occur any time after the gastric operation.[2] When the operation is performed within 48 hours, a mortality of 10% is reported, and as high as 50% mortality can occur when the surgery is delayed beyond 48 hours.[3] The characteristic triad of acute JGI includes sudden onset of epigastric pain, vomiting with or without hematemesis and a palpable epigastric mass (seen in 50% only).[4] The chronic form of JGI is characterized by milder, intermittent symptoms which usually resolve spontaneously. Less than 200 cases of retrograde JGI have been reported,[5] and only one such case has been reported as tumor bleed, to the best of our knowledge.[6]

The aim of our report is to highlight the possibility of JGI as a differential diagnosis of tumor bleed in a gastric surgery patient.

## CASE REPORT

**Figure 1:**
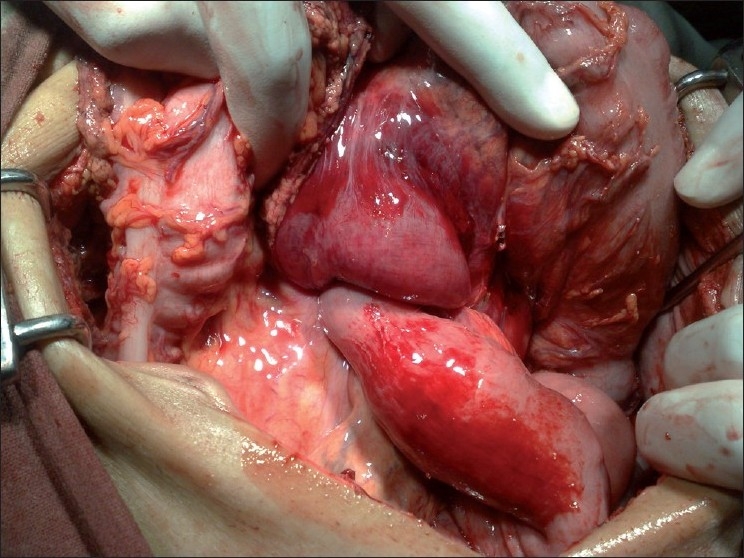
Intussuscepting the efferent jejunal limb into the stomach before gastrotomy incision is made

**Figure 2:**
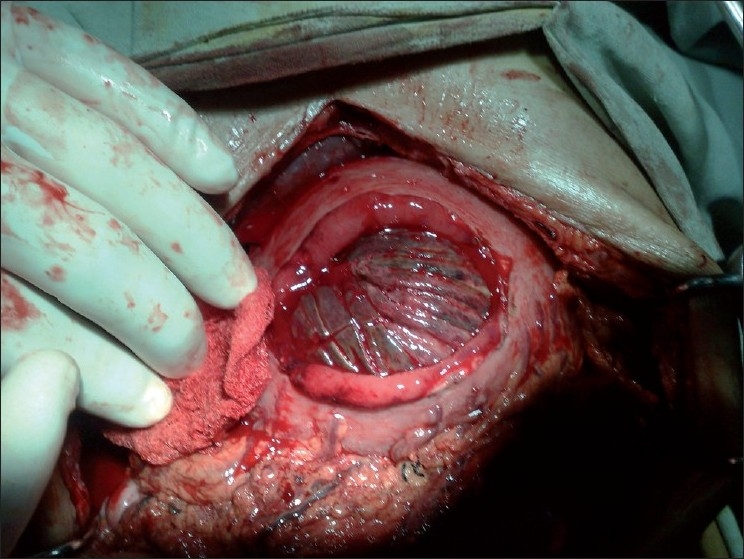
Gangrenous intussuscepted jejunum inside the stomach through gastrotomy incision. The intussusceptum is bleeding and is mimicking a bleeding gastric tumor

**Figure 3:**
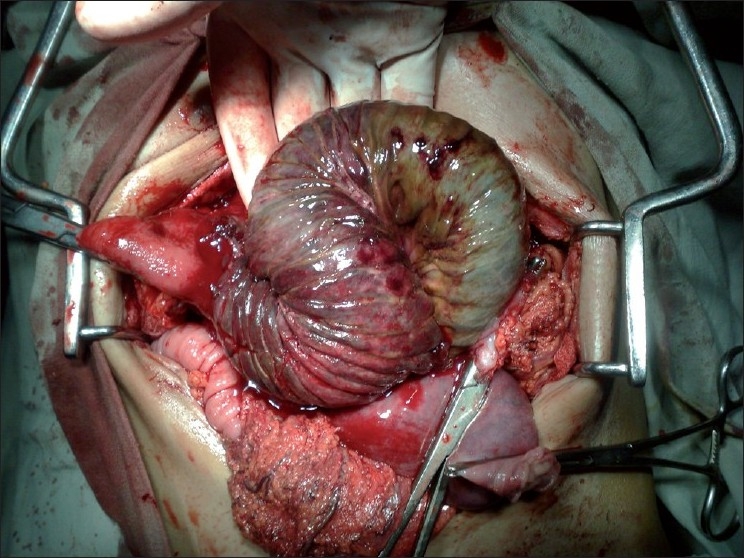
Gangrenous intussuscepting jejunum is delivered out of the gastrotomy incision for resection

A 65-year-old male patient presented to our emergency department with epigastric pain and vomiting for 2 days and hematemesis for 1 day. He had vomited almost 1.5–2 l of blood over the last 24 hours (the vomited blood was preserved by the attendants in a bowl) before presentation. There was history of diarrhea with melina, a sample of which was presented by the attendants in casualty. Previous records revealed that a retro colic gastrojejunostomy with truncal vagotomy was performed 15 years back for peptic ulcer disease. On examination, the patient was pale with a pulse rate of 96 beats per minute (bpm) and blood pressure (BP) of 100/60. A tender, firm epigastric mass was palpable. Laboratory investigations revealed a hemoglobin (HB) of 7.8 g/dl, total leukocyte count (TLC) of 15,000 with 90% neutrophills. Coagulogram, platelet count and serum creatinine were normal. Two large bore intravenous access lines were established, nasogastric tube inserted, and the patient was catheterized. The patient was being resuscitated by crystalloids and blood transfusions to gain time for upper gastrointestinal (GI) endoscopy. X-ray abdomen was done during the process of resuscitation, which was grossly normal. USG abdomen revealed stomach full of echogenic material (blood). After stabilization, the patient was taken for upper GI endoscopy which revealed findings suggestive of a bleeding gastric tumor with stomach full of clots. The bleeding was not controlled by endoscopic measures, and the patient was taken for emergency laparotomy. On laparotomy, efferent limb of jejunum was intussuscepting into the stomach [Figure 1]. Gastrotomy was performed along the greater curvature on the anterior surface of the stomach. The pooled blood and clots were wiped out and the intussuscepted gangrenous efferent jejunal limb was revealed as a mass [Figure 2]. About 25 cm of the gangrenous intussuscepted efferent limb of jejunum [Figure 3] was resected from the stomach, and its stump was closed flush with stomach wall after everting it. Ryle’s tube was passed through antrum, duodenum and back into the stomach through the previous gastrojejunostomy to identify the afferent limb. A fresh anti colic, side to side gastrojejunostomy was performed along the greater curve using the gastrotomy incision. The postoperative period was uneventful and the patient was discharged on 7^th^ postoperative day. Histopathologic examination of the resected bowel showed features of necrosis. He is on our regular follow up and is asymptomatic since the last 6 months.

## DISCUSSION

At the Mayo Clinic in a period of 72 years (1907–1980) years, only 16 well documented cases of JGI have been recognized.[5] Jejunojejunal or jejunoduodenal intussusception has been reported after total gastrectomy, and one case of duodenogastric intussusception has been reported after Billroth I gastrectomy.[5] Shackman described three anatomical variants of JGI. In type I, the afferent loop is intussuscepted into the stomach. In type II, the efferent loop is intussuscepted, and type III represents a combined form. It has been stated that type II is the most common (80%).[7] In our case, a type II JGI was documented on operation, with a long gangrenous jejunal segment inside stomach mimicking a bleeding gastric tumor.

Various factors have been incriminated in the etiology of JGI, such as hyperacidity, long afferent loop, jejunal spasm with abnormal motility, increased intra-abdominal pressure, increased motility of efferent loop, adhesions leading to intussusception of a more mobile segment into fixed segment, widening of upper jejunum, vomiting, pregnancy, labor and other causes of increased intra-abdominal pressure, dilated atonic stomach and retrograde peristalsis.[8] Retrograde peristalsis which can occur in normal people prior to gastric surgery, seems to be accepted as the cause of type II JGI by most authors.[1]

A patient with chronic JGI may present with recurrent episodes of abdominal discomfort which may be associated with nausea that is exacerbated by food, and usually subsides after a couple of hours.[9] In many such patients, the correct diagnosis has never been established. The main reason for this is that upper GI endoscopy must be performed during the symptomatic period, for the diagnosis to be confirmed. However, it has been suggested that in the asymptomatic period, the provocation of JGI during endoscopy by the use of a jet of water directed toward the anastomotic stoma may be diagnostic of the chronic form.[5] Computed tomography shows the classical “target”.[10] However, we could not perform this investigation because the patient was bleeding continuously, and he was taken for emergency laparotomy.

Emergency surgery is the only answer in acute JGI, and various options include reduction of the limb, resection, take-down of the anastomosis, and revision of the anastomosis.[1] Stefano *et al*., in a similar case, tried endoscopic reduction of the intussuscepting limb but did not succeed.[11] Jain *et al*. reported four cases of acute type II JGI. They were able to reduce the intussusceptum in three cases and *in situ* resection of the gangrenous jejunum was performed in one case. They recommended *in situ* resection as the method of choice in case gangrenous intussusceptum is found.[12] We resected the gangrenous limb *in situ* and performed another gastrojejunostomy utilizing the gastrotomy incision. Future recurrence of JGI can be prevented by anchoring the involved jejunal segment to either the neighboring jejunal limb or to the transverse mesocolon.[9]

## CONCLUSION

We conclude that JGI with gangrenous, bleeding intussusceptum can mimic a bleeding gastric tumor, and high index of suspicion regarding JGI is needed in an operated case of gastrojejunostomy or gastrectomy, with a palpable epigastric mass.
